# HIV-1 TAR miRNA protects against apoptosis by altering cellular gene expression

**DOI:** 10.1186/1742-4690-6-18

**Published:** 2009-02-16

**Authors:** Zachary Klase, Rafael Winograd, Jeremiah Davis, Lawrence Carpio, Richard Hildreth, Mohammad Heydarian, Sidney Fu, Timothy McCaffrey, Eti Meiri, Mila Ayash-Rashkovsky, Shlomit Gilad, Zwi Bentwich, Fatah Kashanchi

**Affiliations:** 1 The Department of Microbiology, Immunology and Tropical Medicine program, The George Washington University School of Medicine, Washington, District of Columbia 20037, USA; 2 The Department of Biochemistry and Molecular Biology, The George Washington University School of Medicine, Washington, District of Columbia 20037, USA; 3 Rosetta Genomics Ltd., Rehovot, Israel

## Abstract

**Background:**

RNA interference is a gene regulatory mechanism that employs small RNA molecules such as microRNA. Previous work has shown that HIV-1 produces TAR viral microRNA. Here we describe the effects of the HIV-1 TAR derived microRNA on cellular gene expression.

**Results:**

Using a variation of standard techniques we have cloned and sequenced both the 5' and 3' arms of the TAR miRNA. We show that expression of the TAR microRNA protects infected cells from apoptosis and acts by down-regulating cellular genes involved in apoptosis. Specifically, the microRNA down-regulates ERCC1 and IER3, protecting the cell from apoptosis. Comparison to our cloned sequence reveals possible target sites for the TAR miRNA as well.

**Conclusion:**

The TAR microRNA is expressed in all stages of the viral life cycle, can be detected in latently infected cells, and represents a mechanism wherein the virus extends the life of the infected cell for the purpose of increasing viral replication.

## Background

RNA interference (RNAi) is a regulatory mechanism conserved in higher eukaryotes. RNAi functions through the ability of a small RNA molecule to guide a protein effecter complex to a complementary sequence of nucleic acid [1-3]. The end result is the down regulation of protein expression through either transcriptional silencing, cleavage of target mRNA or inhibition of translation. A key point in understanding RNAi function is the knowledge that a single microRNA (miRNA) may regulate the expression of multiple proteins [2,4]. miRNA is produced from genomic DNA that is transcribed by Pol II in the same manner as mRNA. Hairpin secondary structures in this RNA are recognized and cleaved sequentially by the actions of the Drosha and Dicer enzymes. The resulting miRNA is a duplex of two RNA strands approximately 22 nucleotides in length with a two nucleotide 3' overhang on each strand [4-6]. Ongoing research has revealed that many viruses, including Human Cytomegalovirus, Human Herpesvirus 8, Epstein Barr virus, and Simian Virus 40, express viral miRNA [7-9]. The functions of a limited number of viral miRNA have been determined and they appear capable of regulating both viral and cellular gene expression [9-11].

Human immunodeficiency virus type 1 (HIV-1) is the causative agent of Acquired Immunodeficiency Syndrome (AIDS) [12,13]. Current therapies are capable of controlling viral infection but do not represent a definitive cure. The HIV-1 virus has proven to be capable of developing resistance to therapy, evading the immune response, altering cellular immune function and protecting an infected cell from apoptosis. The virus must accomplish these functions with a limited genome that expresses only nine proteins. As such, the HIV-1 virus must make extensive use of cellular pathways and subvert native molecular processes for its own purpose. Therefore, the inclusion of a miRNA in the viral genome would be a powerful tool for manipulating cellular function [10,14].

We have previously demonstrated the existence of an HIV-1 miRNA derived from the RNA hairpin structure at the 5' end of all HIV-1 transcripts known as TAR [15]. The proteins involved in miRNA biogenesis have been shown to bind to the TAR element and cleavage of TAR by the cellular Dicer enzyme results in the production of a ~22 nucleotide miRNA. This viral miRNA is detectable in infected cell lines, in *de novo *infected primary T-cell blasts, and is detectable throughout the viral life cycle [15]. Previous analyses indicate that this miRNA is functional and may be involved in the regulation of the viral life cycle through suppression of viral transcription. Recently, an independent group has confirmed our findings [16]. At least one paper also suggests that miRNA may be derived from the HIV-2 TAR element, when the HIV-2 TAR is folded in an alternate manner [17]. Here we present the sequence of the HIV-1 TAR miRNA as determined by cloning and show evidence that HIV-1 TAR miRNA alters the expression of a number of important cellular genes. In addition, we show that the outcome of viral miRNA expression is the protection of the infected cell from apoptosis and stress induced cell death.

## Methods

### Cloning and sequencing of the TAR miRNA

cMagi cells were infected with HIV_IIIB _and microRNA enriched libraries were prepared as described using suitable adaptors [18,19]. RT-PCR amplification with an excess of the reverse primer (1:50 ratio) was employed to produce a cDNA library. Biotinylated capture oligonucleotides were then hybridized to an aliquot (5 ul) of the library in TEN buffer. (CTCTCTGGCTAACTAGGGAACCCACTG and ACTGGGTCTCTCTGGTTAGACCAGATTTGA for HIV-mir-3p and HIV-mir-5p respectively) Hybridized pairs were captured by uMACS Streptavidin Kit and the single-stranded miRNA eluted by adding 150 ul of water preheated to 80°C. Recovered single-stranded cDNA molecules were amplified by PCR, ligated into the pTZ57R/T vector and transformed into JM109 bacteria. Positive colonies were identified and sequenced.

### siRNA and RNA molecules

TAR-WT and TAR-D were transcribed from previously described T7 expression vectors [20]. For *in vitro *transcription reactions 1.5 μg of each plasmid was linearized with HindIII (New England Biolabs), ethanol precipitated and used for *in vitro *transcription via the MegaScript High Yield Transcription Kit (Ambion). After transcription TAR RNA was purified on a 2% agarose gel, eluted from the gel with 0.5 M NaAcetate, 1 mM EDTA, 0.2% SDS, and ethanol precipitated before re-suspension in DEPC treated water. siDicer, siLuc, siEGFP and siERCC1 were obtained from a commercial source (Dharmacon). Transfections were performed with Metafectene reagent (Biontex).

### Cells, cell culture and transfections

293T, cMagi, HeLaT4, HLM-1, CEM, ACH2, U1 and U937 cell lines were obtained from the AIDS Reagent program. Adherent cells were cultured in DMEM supplemented with L-glutamine and Pennicilin/Streptomycin with 10% FBS. Suspension cultures were maintained in RPMI-1640 with L-glutamine and Pennicilin/Streptomycin with 10% FBS. For serum starvation experiments, media with 0.1% FBS was used. For transfections, 293T cells were seeded in a 6 well culture plate at 150,000 cells/well. The following day the cells were transfected with 500 ng of the appropriate siRNA or TAR RNA using Metafectene (Biontex) lipid reagent.

### Cell cycle analysis and apoptosis

Cells were washed with PBS and fixed with 70% ethanol. Following rehydration in PBS, cells were stained in PBS containing 25 ug/ml propidium iodide (Sigma), 10 ug/ml RNase A (Sigma) and 0.1% NP-40. Cells were analyzed on a BD FacsCalibur flow cytometer. Cell cycle analysis and measurement of apoptosis was performed using ModFit LT software. Aggregates and debris were excluded by gating on the FL2W and FL2A parameters. Apoptosis was considered to be the population of cells that were sub-G1. Apoptosis analyses were confirmed with BD Biosciences Annexin V Apoptosis detection kit following the procedure outlined by the company.

### Antibodies and Western blots

Dicer antibody was from AbCam. B-actin, Caspase 3, ERCC1, PIASγ, GIT2, p21/waf1 and MDM2 antibodies were from Santa Cruz Biotech. P53 pSer 15 antibody was from cell signaling technologies. Anti-IER3 antibody was a generous gift from Dr. Francoise Porteu, The Cochin Institute, Paris, France. Cell extracts were resolved by SDS-PAGE on a 4–20% tris-glycine gel (Invitrogen). Proteins were transferred to Immobilon membranes (Millipore) at 200 mA for 2 hours. Membranes were blocked with PBS 0.1% Tween-20 + 5% BSA. Primary antibody against either Dicer (AbCam, AB14601) or Actin (SantaCruz, SC-1615) was incubated with the membrane in PBS +0.1% Tween-20 at 0.5 ug/ml overnight at 4°C. Membranes were washed three times with PBS +0.1% Tween-20 and incubated with HRP-conjugated secondary antibody for one hour. Presence of secondary antibody was detected by SuperSignal West Dura Extended Duration Substrate (Pierce). Luminescence was visualized on a Kodak 1D image station.

### Affymetrix MicroArray analysis

RNA samples were submitted to the McCormick Genomics center at the George Washington University Medical for analysis using the Affymetrix Human Focus Array and standard staining and detection procedures. For microarray analysis 293T cells were transfected in triplicate with either TAR-WT, TAR-D or siEGFP. After GC-RMA and normalization TAR-WT experimental values were evaluated as compared to both TAR-D and siEGFP controls. Analysis of variance was performed with a cutoff p-value of 0.05. Expression changes were filtered on a fold change of 1.1 and then grouped according to down or up-regulation. The final list of differentially regulated genes was generated by selecting genes that were similarly regulated in both controls as compared to the TAR-WT experimental transfection (Additional file 1, Figure S1 ).

### RT-PCR

RNA samples were prepared using Trizol reagent (Invitrogen). cDNA was generated using the iScript Select cDNA Synthesis kit (BioRad) according to the manufacturers instructions. Primers used for PCR were: ERCC1F: GGCGACGTAATTCCCGACTA, ERCC1R: AGTTCTTCCCCAGGCTCTGC, IER3F: TCTACCCTCGAGTGGTGAGTATC, IER3R: ACTAAGGGGAGACAAAACAGGAG

## Results and discussion

### Sequencing of the HIV-1 TAR derived miRNA

**Figure 1:**
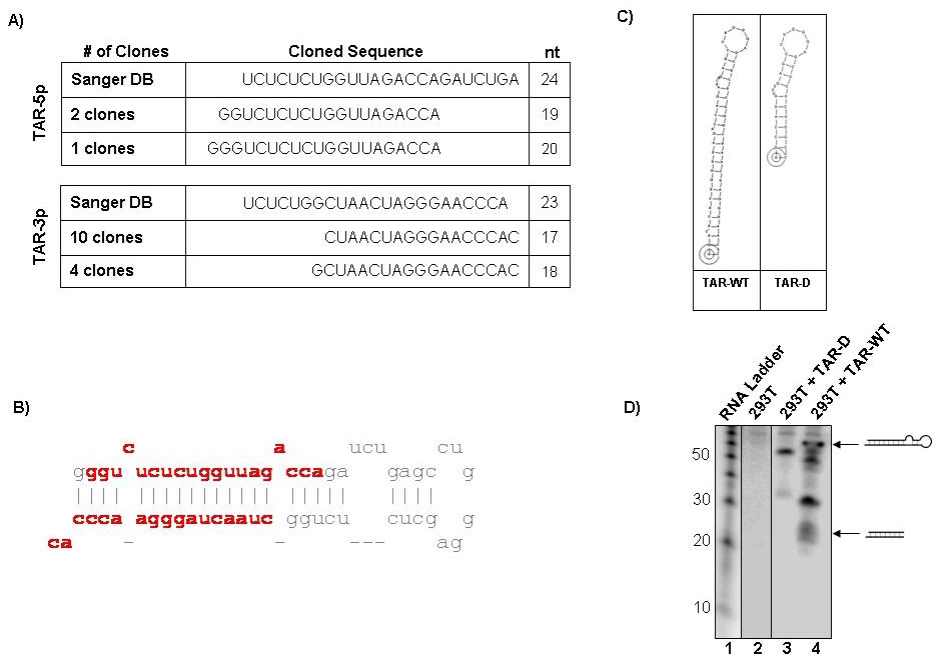
**Determination of the sequence of that HIV-1 TAR miRNA**. RNA from cMagi cells infected with HIV_IIIB _was used to construct miRNA libraries and used for cloning. (**A) **Cloned sequences of the TAR-5p and TAR-3p (5' and 3' arm) miRNA obtained as compared to predicted sequence registered with the Sanger miRNA database. **(B) **Diagram showing the TAR hairpin and the position of the mature miRNA within the TAR sequence. **(C) **Structure of the TAR-WT and truncated TAR-D mutant used for 293T transfections. **(D) **293T cells were mock transfected (lane 2) or transfected with TAR-WT (lane 3) or TAR-D (lane 4) RNA. Forty-eight hours after transfection RNA was isolated and subjected to Northern blotting for TAR sequence. Numbers to the left indicate the size of the RNA ladder in nucleotides. Diagrams to the right show the positions of the wild-type TAR and the mature TAR miRNA.

cMagi cells were infected with HIV_IIIB _and used to prepare microRNA enriched libraries [18,19]. HIV-1 TAR miRNA sequence was then enriched by capture with a biotinylated oligonucleotide. Recovered miRNA library molecules were PCR amplified and cloned into pTZ57R/T vector and sequenced (Fig. 1A and 1B). Cloning analysis recovered three clones of the 5' arm of the TAR miRNA (TAR-5p) and 14 clones of the 3' arm (TAR-3p). The 5' end of the TAR-5p miRNA appears to be defined by transcription start. This and an examination of the 3' miRNA suggest that Dicer acts directly on a short TAR containing hairpin, possibly without any previous Drosha processing. Drosha processing could occur prior to Dicer cleavage and serve to generate the Dicer substrate by freeing the TAR element from a longer RNA. Potential Drosha cleavage may account for the existence of clones starting one base after transcription start (the second G). Comparison to the predicted sequence in the Sanger miRNA database (from Ouellet *et al.*) showed that these cloned sequences differ from previous expectations [16].

### TAR miRNA has an anti-apoptotic effect

We sought to identify a phenotype associated with the TAR miRNA by examining broad effects on the cell cycle. In order to identify the effects of the TAR miRNA specifically, rather than HIV infection in general, we began our investigations by studying 293T cells that were transfected with the TAR RNA. In the first experiment, 293Ts were transfected with either the wild-type TAR RNA (TAR-WT) or with a truncated mutant TAR RNA (TAR-D) (Fig. 1C). Following transfection, RNA extracts were prepared and Northern blotted to confirm the presence of mature TAR miRNA in TAR-WT, but not TAR-D, containing cells (Fig. 1D). Transfected cells were then plated with low-serum media (0.1% FBS DMEM) and harvested after 48 hours. The cells were fixed and stained with Propidium Iodide (PI) and the populations were analyzed by flow cytometry.

**Figure 2:**
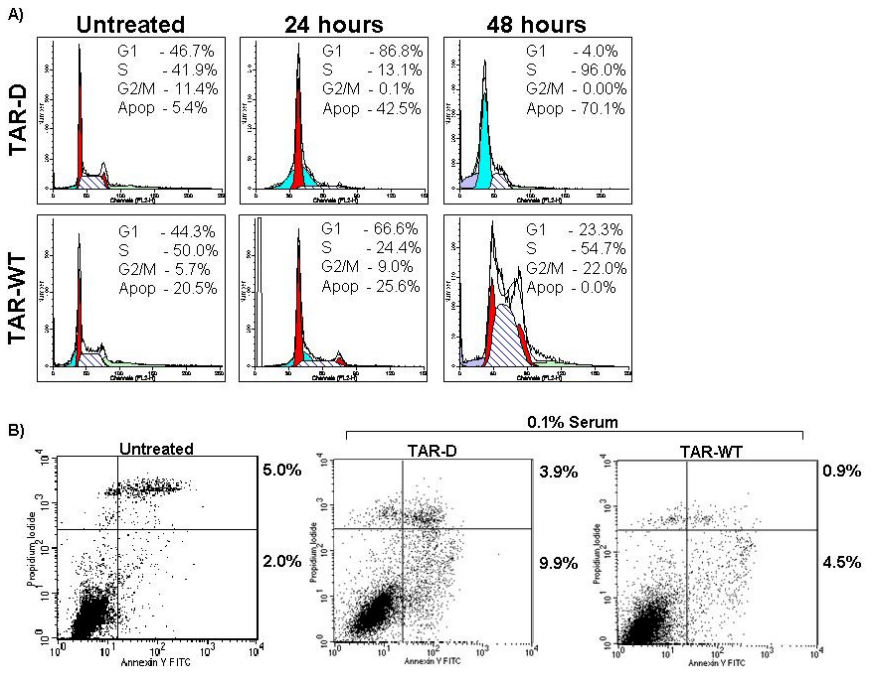
**Transfection of TAR miRNA into 293T cells has an anti-apoptotic effect**. **(A) **293T cells were transfected with TAR-D control or TAR-WT RNA. Twenty-four hours post transfection the media was replaced with DMEM with 0.1% FBS. Cells were sampled at Zero and 48 hours post serum starvation, stained for cell cycle analysis using propidium iodide (PI), and analyzed by flow cytometry. **(B) **HeLaT4 cells were transfected TAR-D or TAR-WT RNA. Twenty-four hours post transfection the media was replaced with DMEM with 0.1% FBS. Cells were sampled at 96 hours, and apoptosis was determined via AnnexinV and PI co-staining. Data are representative of three experiments.

The flow cytometric breakdown indicated that the TAR miRNA had an effect on cell-cycle and survival when under stress. Serum starvation of TAR-D transfected cells led to an arrest in the G1 phase of the cell cycle by 24 hours (86.8% as compared to 46.7% in the cells with full serum). By 48 hours, nearly all the cells were in a sub-G1 peak indicative of possible apoptosis. Whereas the cells without the miRNA showed high levels of apoptosis after 48 hours of serum starvation (70%), the 293T cells with the TAR miRNA showed alterations in cell cycle but were not nearly as apoptotic (no significant change in apoptosis after 48 hours) (Fig. 2A). Interestingly, the TAR-WT containing cells not only survived, but continued to progress through the cell cycle as evidenced by the presence of cells in the S and G2/M phases. Although at 24 hours of serum starvation TAR-WT containing cells did start to accumulate in the G1 phase (66.6% as compared to 44.3%) this did not lead to cell death, and at 48 hours cells were observable in all phases of the cell cycle. The increase in cells in the S-phase as compared to cells with serum suggests that the cells are replicating more slowly. Indeed, TAR-WT transfected cells appear to have a greater portion of cells in the S phase than the control cells even in full serum (compare untreated TAR-D to TAR-WT). The induction of apoptosis was verified using Annexin V staining (2B). HeLa cells were transfected with TAR-D or TAR-WT RNA and serum starved for 96 hours. The increase in Annexin V positive, PI negative cells after serum starvation of the TAR-D transfected cells indicates apoptosis (2.0% to 9.9%). Apoptosis in serum starved, TAR-WT treated HeLa cells was not as high (4.5%). These results suggest that the TAR miRNA is able to decrease levels of apoptosis in stressed cells. To investigate this phenotype in another stress-context, we again used 293T cells transfected with either the TAR-D or TAR-WT, but this time we treated the cells with the DNA crosslinking agent Mitomycin C. Upon analysis by flow cytometry, we observed the same trend as when the cells were deprived of serum; the cells containing the miRNA were more resistant to apoptosis (No increase in the level of apoptosis) compared to the control transfection (TAR-D containing cells experienced over 40 fold increase in apoptosis) (Data not shown). These data indicate that the TAR miRNA has the ability to protect cells from stress-induced cell death.

### Anti-apoptotic effect in infection

After observing the role of the TAR miRNA in protecting the cells from apoptosis under stress, we decided to investigate whether the miRNA had similar effects in chronically infected cell lines. We compared the effects of induced stress on two infected cell lines, HLM1 (HIV-1 infected cervical epithelial carcinoma cell line) and ACH-2 (HIV-1 infected CD4+ T-cell line) to the effects on their uninfected counterparts, HeLa T4 and CEM, respectively. We selected the HLM1 and ACH-2 cell lines as they have often been used as models for viral latency and can be induced to express high levels of viral protein with various agents. We have previously shown that both of these cell lines express the TAR miRNA, by means of an RNase Protection Assay (RPA) with a radiolabeled TAR RNA probe [15]. The four cell lines were plated with 0.1% FBS, and collected daily for four consecutive days. We performed a flow cytometry analysis of the AnnexinV/PI-stained cells in order to determine the possible effects of the TAR miRNA on apoptosis *in vivo*.

**Figure 3:**
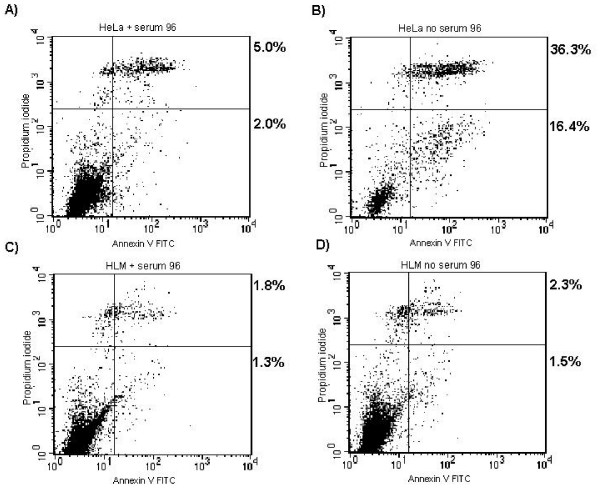
**HIV-1 infected cell lines are resistant to apoptosis**. HelaT4 **(A, B) **and HLM-1 **(C, D) **were cultured in the presence of 10% serum **(A, C) **or 0.1% serum **(B, D) **for 96 hours. Cells were then collected and apoptosis determined via AnnexinV and PI co-staining. Data are representative of three experiments.

According to the FACs analysis, the uninfected HeLa T4s began to apoptose at 48 hours of serum starvation and this continued through 96 hours. The HeLa T4s showed about 16% apoptosis after 96 hours (as compared to only 2.0% in the presence of serum), the HLM1s experienced virtually no increase in the level of apoptosis at the same time point (compare Fig. 3B to 3D). The percentage of PI/AnnexinV double positive cells also increased in the serum-starved HeLaT4 cells and not in the HLM1, suggesting an overall increase in cell death associated with apoptosis.

**Figure 4:**
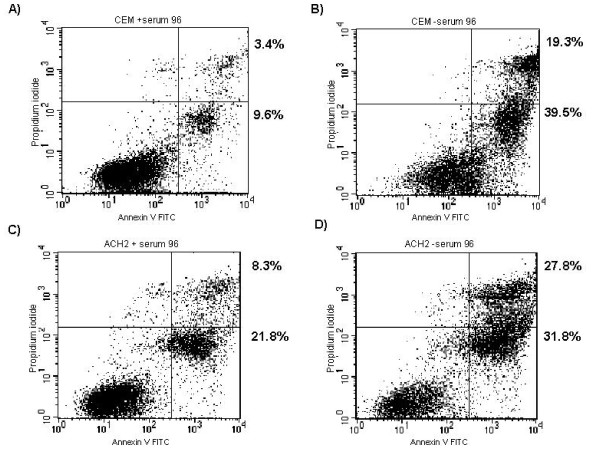
**Infected T-cell lines are resistant to apoptosis**. CEM **(A, B) **and ACH2 **(C, D) **were cultured in the presence of 10% serum **(A, C) **or 0.1% serum **(B, D) **for 96 hours. Cells were then collected and apoptosis determined via AnnexinV and PI co-staining. Data are representative of three experiments.

Like the HLM-1s, the HIV-1 infected ACH-2 cells exhibited a resistance to serum starvation induced apoptosis. When stressed, the levels of apoptosis in ACH-2 cells increased less than in their uninfected control (CEM). According to the flow cytometry analysis of the cell populations, after 96 hours of serum starvation the CEM cells increased in their apoptotic level by 30% whereas the ACH-2 cells increased in apoptosis by only 10% (Fig 4 compare panel B to A and C to D).

**Figure 5:**
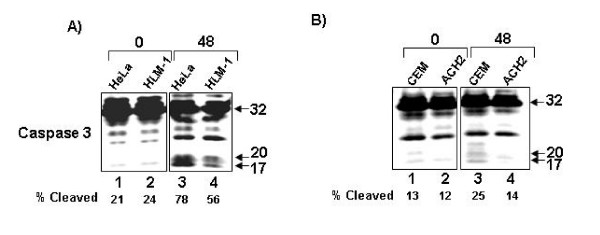
**Serum starvation induced cleavage of Caspase 3 in uninfected but not infected cells**. **(A) **HeLaT4 (lanes 1 and 3) and HLM-1 (lanes 2 and 4) were Western blotted for Caspase 3 expression and cleavage at Zero (lanes 1 and 2) and 48 (lanes 3 and 4) hours after serum starvation. **(B) **CEM (lanes 1 and 3) and ACH2 (lanes 2 and 4) were Western blotted for Caspase 3 expression and cleavage at Zero (lanes 1 and 2) and 48 (lanes 3 and 4) hours after serum starvation. Densitometry was performed to determine the density of the cleaved 17 and 20 kDa Caspase 3 bands as compared to the 32 kDa inactive form.

To confirm this phenotype at the protein level, we Western blotted the extracts from the various cell lines for Caspase 3. The results indicated that at 48 hours of serum starvation, Caspase 3 was cleaved at higher levels in HeLa T4 cells (78% cleavage) than in the HLM1 equivalents (56% cleavage). (Fig. 5A lanes 3 and 4). The increased apoptosis seen at 72 hours is supported by the preceding cleavage of Caspase 3 at 48 hours. Western blotting for Caspase 3 also confirmed that CEM cells are apoptosing at higher levels (25% cleavage) than the ACH-2 cells (14% cleavage) (Fig. 5B, compare lanes 3 and 4). These data indicated that HIV-1 infected cells, which produced detectable levels of the TAR miRNA *in vivo *(and in the case of HLM-1 and ACH-2 produced little to no full length viral mRNA), were capable of withstanding stress-induced apoptosis. The lack of viral protein expression in these cells suggested that this phenotype was due to the viral miRNA processed from short, abortive viral RNA transcripts [21].

### Anti-apoptotic effect is Dicer dependent and can be reversed by blocking miRNA function

**Figure 6:**
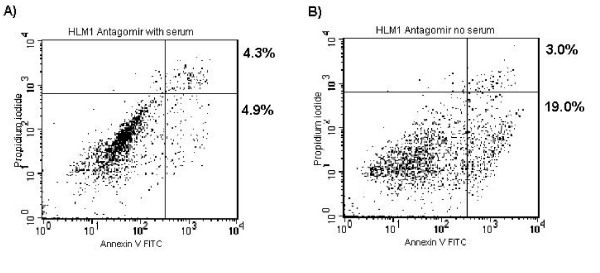
**Specifically blocking TAR miRNA sensitized cells to apoptosis**. HLM-1 cells were transfected with antagomir complementary to the TAR 5' miRNA. Twenty-four hours post transfection the media was replaced with DMEM with 0.1% FBS. Cells were sampled at 96 hours and apoptosis determined via AnnexinV and PI co-staining. Percentages shown indicate the number of AnnexinV positive, PI negative and AnnexinV, PI double positive cells.

As HIV-1 infection or transfection with an RNA may have a broad effect on the cell, we sought to confirm that the anti-apoptotic effect is specific to the TAR miRNA. To test this hypothesis we employed an antagomir, with sequence complementary to the mature miRNA, to prevent the miRNA from functioning. HeLaT4 or HLM-1 cells were transfected with antagomir or were mock transfected. Twenty-four hours after transfection the cells were transferred to low-serum media and grown for 96 hours. Cells were then harvested and apoptosis was determined by AnnexinV/PI staining followed by flow cytometry (Fig. 6). Treatment of the HeLaT4 or HLM-1 cells with antagomir caused no change in apoptosis or cell-cycle progression in the absence of serum starvation (data not shown). HLM-1 cells subjected to mock transfection were still resistant to apoptosis, as we had previously seen (Fig. 6A). However, HLM-1 cells treated with the antagomir showed a level of apoptosis equal to that seen in the HeLa cells (Fig. 6B). The anti-apoptotic effect can be blocked by employing an antagomir specific to the sequence of the TAR miRNA, suggesting that the effect is specific to the miRNA.

To confirm that the anti-apoptotic phenotype is due to miRNA production, and not other viral factors, we knocked down Dicer expression in the HIV-1 infected cells. HLM-1 or HeLa control cells were transfected with siRNA against Dicer (siDicer) or control siRNA (siLuc). Twenty-four hours after transfection the cells were serum starved. At baseline, all four conditions showed comparable levels of apoptosis. At 96 hours of serum starvation, the control HeLa cells showed similar levels of apoptosis regardless of which siRNA was used. However, HLM-1 cells transfected with siDicer showed a level of apoptosis higher than that detected in the HeLa cells (50%) (data not shown). This indicated that resistance to apoptosis was dependent upon the expression of the Dicer protein. As Dicer is required to process the TAR hairpin into a functional miRNA, these results suggest that resistance to apoptosis is mediated by the TAR viral miRNA.

### TAR miRNA alters apoptotic genes

The observation that the HIV-1 TAR miRNA is expressed both in latent and in active infection suggests that the miRNA may play a role in regulating cellular gene expression [15]. We reasoned that expression of the miRNA at all points during infection may have a broad pro-viral effect such as immune evasion, cell survival, or increased viral production. To test this hypothesis, 293T cells were transfected with the TAR-WT (which we have previously shown to be processed into the viral miRNA [15] and Fig. 1D), TAR-D, or a control siRNA (siEGFP) (Fig. 1C).

RNA from the transfection was used for microarray analysis employing an Affymetrix Human Focus Array. Changes in gene expression were considered valid if they occurred in the TAR transfection as compared to both controls, had a P-value of less than 0.05, and the levels of detection changed by more than 10% (Fig S1). This analysis indicated that 32 genes were significantly altered by the presence of the HIV-1 miRNA (18 down-regulated, 14 up-regulated). As the primary function of RNAi is to silence gene expression, we postulated that the up-regulated genes may be a secondary effect related to repression of a regulatory gene. After examining the down-regulated genes we identified many potentially interesting targets related to replication, receptor signaling, DNA repair, mitochondrial function and apoptosis. In order to determine which of these pathways was truly regulated by the viral miRNA we sought to determine which genes may be related to the observed phenotype.

**Figure 7:**
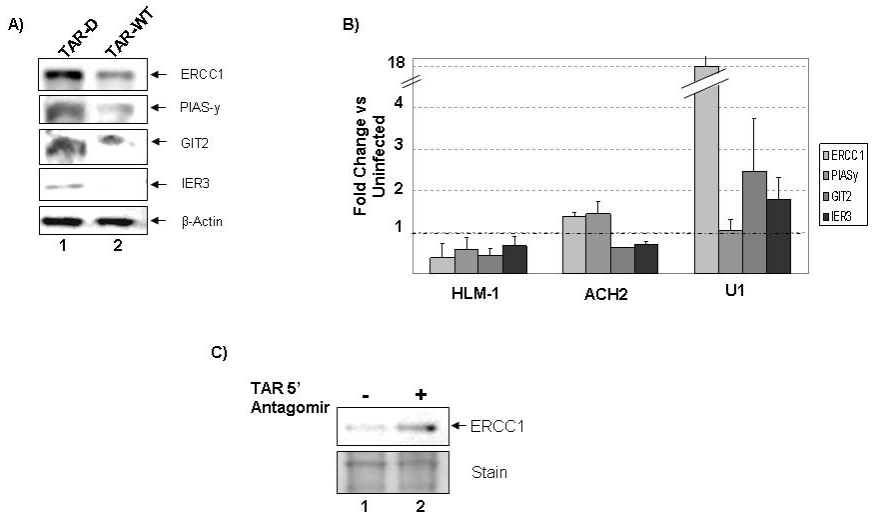
**HIV-1 miRNA down-regulated the expression of proteins related to apoptosis**. **(A) **293T cells were mock transfected (lane 1) or transfected with TAR-D mutant (lane 2) or TAR-WT RNA (lane 3). After 48 hours cell lysates were prepared and Western blotted for ERCC1, PIASγ, GIT2, IER3 or β-actin. **(B) **Cell lysates were prepared from HeLaT4, HLM-1, CEM, ACH2, U1 and U937 cell lines and Western blotted for ERCC1, PIASγ, GIT2, IER3 or β-actin. Densitometry was performed, and the expression levels were normalized to actin. The average expression level of each protein from three experiments was determined and displayed as the ratio of expression in the infected cells (HLM-1, ACH2 and U937) to their uninfected counterpart (HeLaT4, CEM, U1). **(C) **HLM-1 cells were transfected with mock (lane 1) or TAR 5' antagomir (lane 2). Cells were lysed after 96 hours and 20 micrograms of protein were used to Western blot for the expression of ERCC1. Coomassie staining of the ~25–50 kDa portion of the gel is included as a loading control.

In examining the potential list of HIV-1 miRNA regulated genes, we selected four genes with possible links to apoptosis and cell survival for further study; ERCC1, PIASγ, GIT2 and IER3. Excision repair cross complementing-group 1 (ERCC1) is involved in the detection and base excision repair of damaged nucleotides [22]. Protein inhibitor of activated STAT Y (PIASγ) is an inhibitor of STAT1 signaling, and is capable of modulating NFκB signaling, and also functions as a transcriptional co-repressor due to E3 Sumo ligase activity [23,24]. G protein-coupled receptor interacting protein (GIT2) is involved in G-protein signaling [25]. Intermediate early response 3 (IER3) is up-regulated after cellular insult and has been shown to be required for induction of apoptosis after serum starvation and DNA damage [26-29]. We tested the ability of the TAR miRNA to down-regulate these four genes using Western blotting (Fig. 7A). 293T cells were transfected with TAR-WT, TAR-D or were mock transfected. Forty-eight hours after transfection, cell extracts were prepared, and protein expression was examined by Western blotting. While there was no change in the expression level between mock and TAR-D transfections, ERCC1, PIASγ, GIT2 and IER3 were all down-regulated in the presence of TAR-WT when normalized to actin.

To confirm that these proteins were differentially regulated in infected cells, Western blottings were performed on the infected cell pairs: HeLa/HLM-1, CEM/ACH2 and U1/U937 (Fig. 7B). Expression was quantified as the ratio of protein levels in HLM-1, ACH2 and U1 as compared to their uninfected control. U1 and U937 were included as controls in this experiment because U937 cells do not express Dicer [15] and hence they should not show differential expression of the target genes. All four genes of interest were down-regulated in HLM-1 as compared to HeLa (ranging from 30–60% decrease). GIT2 and IER3 were down-regulated in ACH2 as compared to CEM, while ERCC1 and PIASγ were not significantly changed. PIASγ expression was also not altered in U1 as compared to U937. Interestingly, the expression levels of ERCC1, GIT2 and IER3 were increased in U1 as compared to the uninfected U937 cells. This indicated that the viral infection may be up-regulating expression of these proteins, and the viral miRNA was serving to counteract this effect. Collectively, these experiments indicated that HIV-1 miRNA down-regulated ERCC1, GIT2 and IER3.

To verify that repression of ERCC1 in the infected cells was due specifically to the action of the miRNA, and not other viral factors, we again employed the antagomir specific for the TAR 5' miRNA (Fig. 7C). HLM-1 cells were transfected with TAR 5' antagomir or were mock transfected. Four days after transfection the cells were collected, lysed, and proteins were separated by SDS-PAGE and Western blotted for ERCC1. Transfection of the antagomir increased the level of detectable ERCC1 in HLM-1 cells.

**Figure 8:**
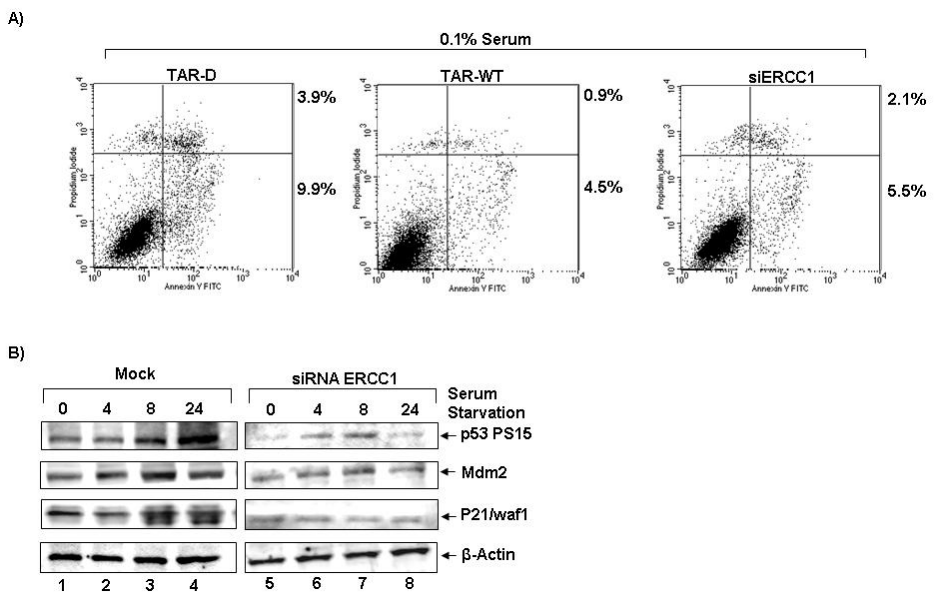
**Anti-apoptotic effect validated through the repression of ERCC1**. **(A) **HeLaT4 were transfected with TAR-D, TAR-WT, siEGFP, or siERCC1 RNA. Twenty-four hours after transfection media were replaced with low serum (0.1%) media, and the cells were cultured for 96 hours. Apoptosis was measured at 96 hours after serum starvation using FACs analysis. Data are representative of two experiments. **(B) **293T cells were transfected with siRNA against ERCC1 (lanes 5–8) or mock (lanes 1–4) for twenty-four hours, and then the media was replaced with low serum media. Cells were harvested for Western blot analysis at 0, 4, 8 and 24 hours after serum starvation and Western blotted for phosphor-p53 Ser15, Mdm2, p21 and β-actin. Pictured Western blots utilized 20 micrograms of total protein per lane.

ERCC1 was upregulated by viral infection in the absence of Dicer by 18 fold (Fig. 5B). This is in keeping with published reports that viral infections, including HIV-1, up-regulate the expression of DNA repair proteins. ERCC1 is involved in the recognition and repair of DNA damage. Indeed, previously published reports indicate that increased levels of ERCC1 correlate with resistance to DNA damage induced apoptosis [30-32]. Our findings suggest a novel role for ERCC1 in inducing apoptosis in response to serum starvation. To confirm the role of ERCC1 in protection from serum starvation induced apoptosis, siRNA was utilized. 293T cells were transfected with TAR-D, TAR-WT or siRNA against EGFP or ERCC1. Cells were serum starved for 48 hours, and the level of apoptosis was determined at 96 hours post serum starvation (Fig. 8A). Control transfection of TAR-D showed that 9.9% of the cells were apoptotic. siRNA against ERCC1 prevented the induction of apoptosis at 48 hours, comparable to the transfection with wild type TAR RNA. Transfection of 293T cells and cell cycle analysis confirmed these results (data not shown). These results suggested that in the setting of 293T cells, repression of ERCC1 expression inhibited apoptosis triggered by serum starvation. IER3 has previously been shown to be involved also in serum starvation induced apoptosis [26-29]. Together these data suggested that the TAR miRNA prevented apoptosis by down-regulating both ERCC1 and IER3.

Induction of apoptosis via serum starvation is mediated by p53. Activation of p53 induces the expression of Mdm2, p21/waf1 and Bax [33]. Bax is trans-located to the mitochondria and begins the apoptotic cascade [34]. Mdm2 and p21/waf1 serve to regulate the cell cycle and feed back on p53 [33,35]. We sought to confirm the involvement of ERCC1 repression by the TAR miRNA in p53 mediated apoptosis by following the activation state of p53 and the expression of Mdm2 and p21/waf1 (Fig. 8B). 293T cells were mock transfected or transfected with siRNA against ERCC1. Twenty-four hours after transfection the cells were cultured in 0.1% FBS for 0, 4, 8 and 24 hours. Cell extracts were prepared at each time point and Western blotted for p53 phosphorylated on serine 15 (p53 PS15), Mdm2, p21/waf1 and β-actin. Serum starvation of the control cells triggered an increase in p53 phosphorylation and a subsequent increase in the expression of Mdm2 and p21/waf1 (Fig. 8B, lanes 1–4). The phosphorylation of p53 was highest at the 24 hour time point, but increased phosphorylation can be seen from 4 hours on. Interestingly, 293 cells transfected with the siRNA against ERCC1 showed only a small increase in p53 phosphorylation that peaked at 8 hours (Fig. 8B lane 7), a slight increase in Mdm2 at 8 hours and no increase in p21 expression (lanes 5–8). Furthermore, the levels of p53 phsophorylation, Mdm2 and p21 in the ERCC1 knockdown before serum starvation all appeared lower than in the control cells (compare lane 1 to lane 5). Transfection of TAR-WT RNA showed a similar phenotype as the ERCC1 knockdown (data not shown). These results indicated a new role for ERCC1 in influencing the activation of p53 in response to cellular stress.

**Figure 9:**
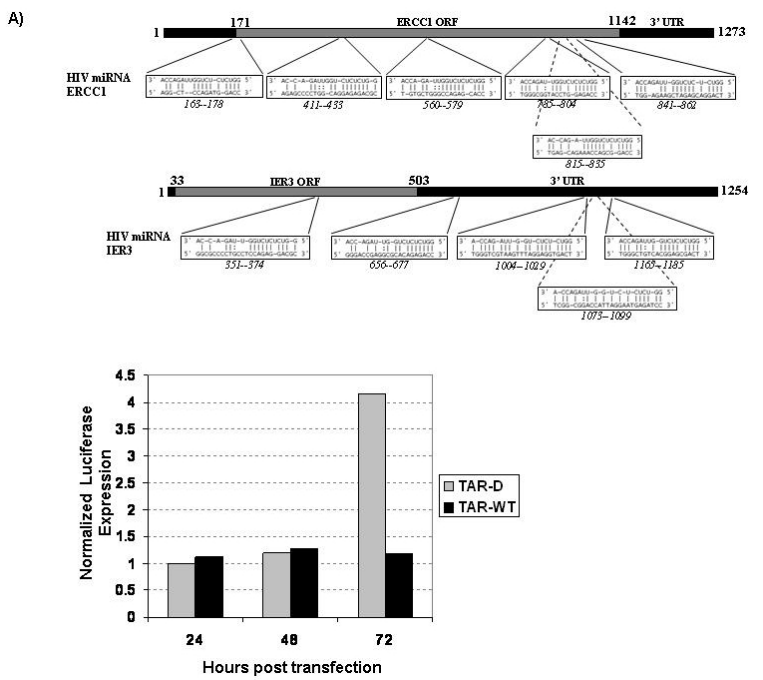
**TAR 5' miRNA targeted the ERCC1 gene directly**. **(A) **miRanda software was used to determine potential target sites in the ERCC1 and IER3 mRNA sequences for the HIV-1 TAR 5' miRNA. **(B) **293T cells were transfected with TAR-D or TAR-WT RNA. Twenty-four hours after RNA transfection the cells were transfected with psiCheckERCC-737 reporter vector. Extracts were prepared and luciferase expression determined at 24, 48 and 72 hours after reporter transfection. Data shown represent the normalized expression of *Renilla *luciferase (with the target region) to firely luciferase. Data are representative of two replicates.

Repression of gene expression by RNAi requires sequence homology between the target and the miRNA. We analyzed ERCC1 and IER3 mRNA sequences for the presence of sequences complementary to the HIV-1 viral miRNA using the search algorithm miRanda (Fig. 9A) [36]. Statistical analysis was performed to eliminate matches due to random chance. Six potential target sequences were identified within the ERCC1 mRNA and five within the IER3 mRNA. Analysis also indicated that each of the genes listed in Table 1 as being down-regulated, with the exception of TIMM, have multiple target sites for the viral miRNA. Interestingly, multiple target sites for the TAR 5' miRNA were found in 17 of the 18 down-regulated genes (Additional file 2, Supplemental file 2). However, the TAR 3' miRNA had only single possible targets in a small number of genes (Additional file 3, Supplemental file 3). This suggests that the TAR 5' miRNA is more likely the functional RNA strand.

To confirm that TAR derived miRNA can target the ERCC1 gene we cloned a cluster of the potential target sites into the psiCheck reporter construct (Invitrogen) (Fig. 9B). psiCheck contains a *Renilla *luciferase reporter with a multiple cloning site in the 3'UTR for the insertion of potential miRNA target sites, and a firefly luciferase reporter for normalization. A region of the ERCC1 gene containing three potential target sites (bp 737–945) was PCR amplified and inserted into the psiCheck vector to form psiCheckERCC-737. 293T cells were transfected with either TAR-D or TAR-WT RNA. Twenty-four hours after transfection of RNA the psiCheckERCC-737 vector was transfected into the cells. Cell extract was prepared at 24, 48 and 72 hours after transfection. Luciferase expression was determined, and the expression of the target site containing *Renilla *luciferase was normalized to the firefly luciferase. No expression of *Renilla *luciferase was detected on day one or two. However, at 72 hours, robust luciferase expression was detectable in the TAR-D containing cells and not the TAR-WT cells (Fig. 9B). Neither TAR-D nor TAR-WT had any effect on the expression of a reporter not containing the target sequence (data not shown).

### TAR miRNA altered the protein expression without affecting the mRNA

**Figure 10:**
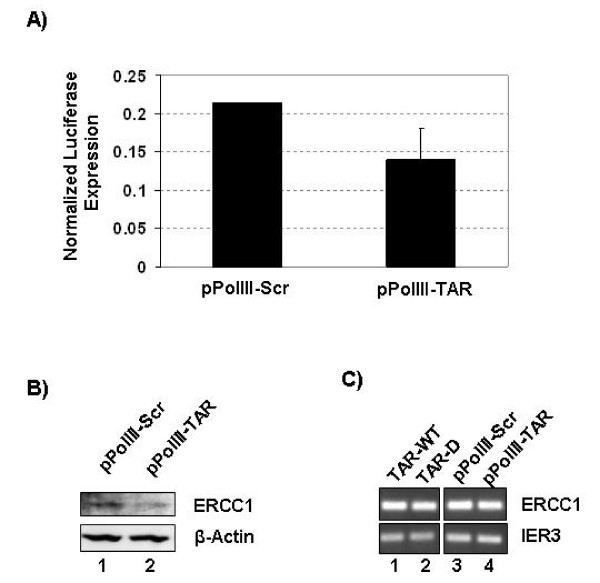
**TAR miRNA altered ERCC1 protein expression without altering mRNA levels**. **(A) **293T cells were transfected with psiCheckERCC-737 and either pPolIII TAR or pPolIIIScr. Renilla and firefly luciferase expression was measured after 72 hours. Data shown represent the normalized ratio of Renilla luciferase to firefly luciferase for two replicates. 293T cells were transfected with either pPolIII TAR or pPolIIIScr. Forty-eight hours after transfection the cells were harvested and protein and RNA extracts were prepared. **(B) **Protein extracts were Western blotted for ERCC1 and β-actin expression. **(C) **RNA extracts were used to generated cDNA, and ERCC1 and IER3 mRNA levels were determined by PCR.

Our results indicated that the TAR 5' miRNA repressed ERCC1 expression and cellular apoptosis. Presumably this effect is through the miRNA pathway; perhaps through the silencing of translation by recruitment of the mRNA to the P-body. To confirm this assumption, we sought to examine the level of mRNA expression of ERCC1 and IER3 in the presence and absence of the TAR miRNA. To further control for a miRNA effect, we sought to utilize a control that still produces a mature miRNA, but has a mutated seed sequence. We used a pair of Pol III expression vectors that express either a WT TAR or a TAR element with a scrambled sequence in the stem corresponding to positions 6–16 of TAR and the complementary based on the 3' side of the stem (Generous gift of Dr. Rossi, City of Hope, CA). Both the pPol III-TAR and pPol III-Scr vectors produce a mature miRNA (Rossi and Castanotto, unpublished data). To verify that Pol III-TAR produces a miRNA that can affect ERCC1, we again performed the luciferase reporter assay using the psiCheck and psiCheckERCC-737 vectors (Fig. 10A). 293T were transfected with psiCheck or psiCheckERCC-737 and pPol III-Scr or pPol III-TAR vectors, and luciferase expression was measured 72 hours later. Renilla expression was normalized to an internal control firefly luciferase. The data indicated that pPol III-TAR vector suppressed the expression of the luciferase mRNA containing the miRNA target sequence. This effect was not seen with a control vector (data not shown).

We next sought to examine the ability of pPol III-TAR to effect ERCC1 protein expression and mRNA levels. 293T cells were transfected with pPol III-TAR or pPol III-Scr. Forty-eight hours after transfection cells were harvested and protein and RNA extracts prepared. Protein extracts were analyzed by Western blotting for the expression of ERCC1 and β-actin (Fig. 10B). The Western blot analysis revealed the repression of ERCC1 protein expression by the pPol III-TAR plasmid, similar to the results seen with the transfection of the TAR-WT RNA. To confirm that this repression was due to miRNA and not to any secondary effect of the procedure, RT-PCR was performed on the extracted RNA to examine the mRNA levels of ERCC1 and IER3 (Fig. 10C). PCR results indicated no change in the levels of mRNA for either gene. RT-PCR on RNA extracts from 293T cells transfected with TAR-D or TAR-WT RNA also indicated no change in mRNA level. These results strongly suggested that the TAR miRNA worked by repressing the translation of targeted mRNA.

## Conclusion

Using a modified version of the standard miRNA sequencing protocol, we enriched HIV-1 TAR derived miRNA using biotinylated capture oligonucleotides. This approach allowed us to successfully obtain the exact sequence of both strands of the TAR miRNA. Interestingly, we were able to sequence more clones for the 3' miRNA (hiv1-miR-TAR-3p) than the 5' miRNA (hiv1-miR-TAR-5p). However, the identification of target sites in ERCC1 and IER3 revealed a greater number of target sites for the TAR-5p than the TAR-3p. This trend was also found for the other genes identified as down-regulated in our microarray experiment (with the exception of Translocase of inner mitochondria membrane which contains no target sequences, Supplemental Data). These findings are in keeping with our previous work, wherein we could detect the 5' miRNA sequence, but not the 3'sequence in stably infected cell lines.

The cloning described in this current work revealed a different sequence for the TAR 5' than what has been previously proposed (Fig. 1A). Our cloning analysis suggested the existence of a 20 nucleotide product starting from transcription start site. Previous work by Ouellet *et al. *predicted a 24 nucleotide miRNA that starts from +4 after the transcription start site [16]. The assays presented in our manuscript lead us to believe that our sequenced clone is correct. However, we cannot rule out the alternative form since the important seed sequence for both clones overlap and may allow them to target similar mRNAs. Further work must be performed to distinguish which of the two miRNA RNA is expressed in HIV-1 infected cells and which is functional.

The HIV-1 TAR miRNA causes cells to become resistant to apoptosis in the setting of transfection and infection, and this effect is dependent upon Dicer expression. These data indicate that the HIV-1 TAR miRNA is capable of down-regulating cellular gene expression and altering the cellular phenotype. The viral miRNA is expressed at all stages of the viral life cycle and thus has implications for HIV-1 infection [15]. Previously, HIV-1 infection has been shown to alter cellular gene expression [37-39] and cellular microRNA expression [40-42]. These changes are mediated by viral proteins, with a particular emphasis on Tat, Nef and Vpr. The alteration of cellular gene expression by viral protein has been linked to evasion of the immune response (downregulation of MHC by Nef), resistance to apoptosis (Nef), induction of apoptosis in bystander cells (Tat and VPR), alterations in cell cycle and replication (Tat) [39,43-45], and suppression of RNAi silencing (Tat) [46-48]. All of these changes are meant to increase viral replication and prolong the period of time in which a cell is capable of producing infectious virus. Our data on the HIV-1 viral miRNA, however, demonstrates a role for viral RNA in altering cellular gene expression. These findings are in keeping with previous reports on alteration of cellular pathways by the virus, as the miRNA serves to protect the cells from apoptosis. These data take on even greater significance due to the fact that the HIV-1 miRNA is expressed in active and latently infected cells – even in the absence of viral protein production.

We performed microarray analysis to screen for genes possibly regulated by the HIV-1 TAR miRNA. Although miRNA are generally active in the repression of translation, we took the relatively modest changes in mRNA as a hallmark of possible silencing of protein translation. To confirm this hypothesis we examined the expression of four proteins with possible links to apoptosis; ERCC1, PIASγ, GIT2 and IER3 [22-26]. Although all four genes were down-regulated in transfection, only GIT2 and IER3 were reliably down-regulated in HLM-1 and ACH2 as compared to their control. Interestingly, the levels of the ERCC-1 protein seem to be drastically up-regulated during infection in the absence of functional RNAi. This raises the possibility that the miRNA may be specifically acting to prevent changes in gene expression caused by viral infection. Indeed, preliminary experiments indicate that the mRNA levels of ERCC1 are elevated in ACH2 cells as compared to CEM, despite the opposite being true at the protein level (data not shown and Fig. 7). Although GIT2 has potentially important roles in regulating cell survival, the silencing of IER3 and ERCC1 are likely the reason for the anti-apoptotic phenotype we have observed. IER3's effect on apoptosis has been previously studied. Interestingly, in the setting of serum starvation and DNA damage, the loss of IER3 expression is anti-apoptotic [27-29]. Our finding that cells containing the HIV-1 miRNA are protected from MMC induced apoptosis (Fig. 2) supports a role for the miRNA in regulating IER3. Further analysis indicates that in 293T cells the silencing of ERCC1 by siRNA is sufficient to protect the cells from serum starvation induced apoptosis. Our previous work indicated that the viral miRNA may also function to regulate viral gene expression: an effect similar to the miRNA encoded by SV40 and involved in viral latency [9,15].

We found the presence of multiple validated target sites in the ORF of the ERCC1 gene intriguing. The generally accepted rules for miRNA targeting require a rather precise seed sequence match to an area in the 3'UTR. However, in this case, the HIV 5' miRNA appears to target a triplex of sites in the reading frame of the gene. Cloning of this region into a reporter indicates that it is a functional target and the presence of three closely located sites also suggests a bona fide miRNA target. The lack of perfect base-pairing in the seed sequence is reminiscent of that described for the let-7:lin-41 interaction, wherein a single base bulge is tolerated in the miRNA:target pairing [49]. Work on miRNA targeting suggests that the ability of a miRNA to successfully repress a target gene is very dependant on the context of the sequence flanking the target sites [50-52]. Several other examples, including the targeting of Dicer by let7 and microRNAs regulating Nanog, Oct4 and Sox2, indicate that microRNAs are capable of down-regulating genes through targeting of their ORFs [53,54]. One group even suggests that miRNA can target the 5'UTR [55]. We believe that this work has uncovered another miRNA capable of driving down-regulation of a target gene through interaction with a sequence in the ORF. These findings serve to indicate that there is still much that we do not know about how miRNA functions and indicate a need for ongoing research into how the RISC complex interacts with target mRNA.

A recent trend in the development of HIV-1 therapeutics has been to use peptides and small molecules in an attempt to specifically induce cell death in the HIV infected cell [56,57]. This approach has become attractive as it would clear the reservoir of cells producing virus, including the latent pool [58]. The discovery that infected cells which are transcriptionally silent may still be resistant to apoptosis due to the presence of TARmiRNA has clear implications for therapy.

On the basis of these findings, we propose the following model for the action of HIV-1 TAR miRNA. Basal transcription of the HIV-1 LTR leads to production of short, TAR containing, RNA hairpin sequences. These hairpins are acted upon by the proteins involved in miRNA biogenesis, specifically Dicer to yield a viral miRNA. This miRNA is loaded into the RISC complex and regulates the expression of several cellular genes through inhibition of translation, leaving mRNA levels un-affected. The net effect of this interference is that the infected cell becomes resistant to apoptosis. Previous studies on the IER3 gene suggest that it may be involved, but our analysis indicates that another major target of the HIV-1 viral miRNA may be the ERCC1 gene.

## Competing interests

The authors declare that they have no competing interests.

## Authors' contributions

ZK conceived of the experiments, wrote the manuscript and aided in the RNA and protein studies. RW performed the Western blots, cell cycle and cell culture assays. JD and RH performed Western blot analyses. LC performed the p53 analysis. MH, SF and TM performed and oversaw the microarray experiments. EM, MA, SG and ZB performed the cloning and sequencing of the TAR miRNA. FK oversaw the research and aided in the preparation of the manuscript.

## Supplementary Material

Additional file 1 **Figure S1**. Schematic representation of the interpretation of the microarray results. Click here for file

Additional file 2 **Supplemental file 2**. Raw output data from miRanda analysis. TAR 5' miRNA sequence was used to search for targets within the genes indicated as down-regulated by the microarray. Click here for file

Additional file 3 **Supplemental file 3**. Raw output data from miRanda analysis. TAR 3' miRNA sequence was used to search for targets within the genes indicated as down-regulated by the microarray. Click here for file
