# Supplementary figures and images for: HIV-1 TAR miRNA protects against apoptosis by altering cellular gene expression
Source: Retrovirology. 2009 Feb 16;6:18. doi: 10.1186/1742-4690-6-18 (PMC2654423; doi:10.1186/1742-4690-6-18)

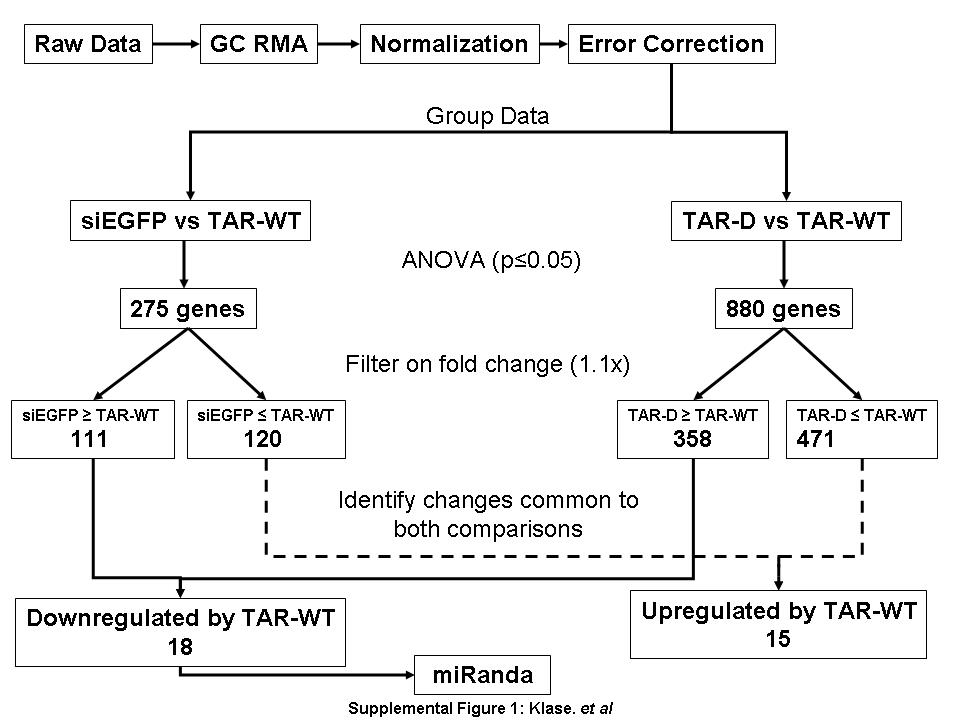

Supplement: Additional file 1 — Figure S1. Schematic representation of the interpretation of the microarray results. [file 1742-4690-6-18-S1.jpeg]
